# ^177^ Lu-based radioligand therapy: A retrospective multicenter analysis to calculate the effective half-life and follow-up dose for the public

**DOI:** 10.1055/a-2784-7468

**Published:** 2026-01-29

**Authors:** Christian Schütze, Markus Borowski, Martin Freesmeyer, Robert Freudenberg, Heribert Hänscheid, Jens Kurth, Marc Rudi Linde, Lara-Christiane Militzer, Bernhard Sattler, Tabea Nikola Schmidt, Paul Soubiran, Mathias Steffner, Lars Stegger, Carsten Wanke, Christian Kühnel

**Affiliations:** 1 27177 Clinic for Nuclear Medicine, University Medical Center Göttingen, Göttingen, Germany; 2 Institute for Radiology and Nuclear Medicine, Braunschweig Municipal Hospital, Braunschweig, Germany; 3 39065 Clinic for Nuclear Medicine, Jena University Hospital, Jena, Germany; 4 39063 Clinic and Polyclinic for Nuclear Medicine, University Hospital Carl Gustav Carus, Dresden, Germany; 5 Clinic for Nuclear Medicine, University Hospital Würzburg, Würzburg, Germany; 6 39071 Clinic for Nuclear Medicine, University Hospital of Rostock, Rostock, Germany; 7 9231 Institute for Nuclear Medicine, Vivantes Hospital Group, Berlin, Germany; 8 39066 Clinic and Polyclinic for Nuclear Medicine, University Hospital Leipzig, Leipzig, Germany; 9 9177 Department of Radiation Protection and Medical Physics, Hannover Medical School, Hanover, Germany; 10 39069 Clinic for Nuclear Medicine, University Hospital Münster, Münster, Germany

**Keywords:** ^177^ Lu radioligand therapy, effective half-life, Radiation protection, Dose rate measurement, PSMA, RRT

## Abstract

The study investigated routinely used radiopharmaceuticals containing lutetium-177 ( ^177^ Lu), providing reference values for their effective half-life (T _1/2,eff_ ). So far, no guidelines regarding discharging this patients exist, are essential to ensure radiation protection of the public. This study contributes to the development of binding discharge criteria regarding ^177^ Lu therapies.

This retrospective multicenter study comprised eight nuclear medicine departments. Two commercially available products and two in-house preparations were considered. Radiation measurement methods, in terms of measuring device, setup, and time points were reported, as well as dose rate measurements for T _1/2,eff_ calculation.

The study includes 210 dose rate measurement sets with a mean administered activity of (7149 ± 522) MBq. When only measurements up to 48 h p.i. were taken into account the T _1/2, eff_ were shorter (Pluvicto 1.30 d, Lutathera 1.40 d, ihPSMA 1.45 d, ihRRT 1.97 d) compared to 168 h (1.60 d, 1.89 d, 2.10 d, and 2.85 d, respectively). Differences were statistically significant for all compounds except Lutathera. The 90 ^th^ percentile of the T _1/2,eff_ observed in late measurements (Pluvicto 2.0 d, Lutathera 3.0 d, ihPSMA 3.5 d, ihRRT 4.0 d) can be considered conservative values for discharge calculations.

The T _1/2,eff_ of ^177^ Lu is considerably shorter than its T _1/2,phy_ , owing to the pharmacokinetics of the entire molecule. This highlights the need of considering biological clearance mechanisms into calculations of discharge times for patients treated with ^177^ Lu. If direct measurements are not feasible in clinical routine, reference values support inpatient planning while ensuring radiation protection.

## Introduction

Radioligand therapies using lutetium-177 ( ^177^ Lu)-labelled compounds have become well-established therapies in nuclear medicine. They are particularly important in the management of metastatic castration-resistant prostate cancer using prostate-specific membrane antigen (PSMA) ligands, and in the treatment of neuroendocrine tumors with peptide receptor radionuclide therapy (RRT) with somatostatin analogues 1 2 . However, despite their widespread clinical use, discharge criteria following administration and the half-lives administered for dose estimation remain inconsistently defined in Germany 3 4 .

According to §122(4) of the German Radiation Protection Ordinance (Strahlenschutzverordnung, StrlSchV), the release of patients from nuclear medicine wards is only permissible if the expected effective dose to members of the public, including relatives and caregivers, does not exceed 1 millisievert (mSv) per calendar year. This limit must be considered cumulatively across all radionuclide therapies administered within a given year.

For thyroid therapies with iodine-131 (131I), which are commonly administered orally using capsules, discharge criteria are outlined in the German guideline “Radiation Protection in Medicine” 5 6 . These stipulate a minimum inpatient stay of 48 h and give a conservative estimate for the dose rate at patient discharge of 3.5 µSv/h at a distance of 2 m. These criteria are based on conservative assumptions, namely, the continuous presence of another individual at a distance of 2 m until complete physical decay of the administered activity, applying the T _1/2, phy_ for 131I (8.02 d). Importantly, a significant fraction of the administered activity is excreted within the first 48 h after administration 7 . Excretions are collected and safely managed via the hospital’s internal decay system 8 . The majority of thyroid disease patients are not treated more than once per calendar year, thus this criterion does not result in an increased length of stay.

Currently, no fixed discharge criteria are defined for intravenously administered ^177^ Lu-therapies, although these procedures have become standard in recent years 9 10 . The only available recommendation was published by the German Radiation Protection Commission and relies on the T _1/2, phy_ of ^177^ Lu; however, following this conservative approach could suggest exceedances if multiple administrations are performed within one year. Since only a few studies have addressed this topic to date, robust clinical data are scarce. Therefore, the T _1/2, phy_ remains a provisional parameter for defining discharge conditions 11 12 13 . Since ^177^ Lu-therapies are administered in repeated cycles rather than as single administrations, discharge criteria must account for cumulative exposures across treatment courses 14 . Current clinical guidelines recommend up to six cycles of PSMA-targeted therapy or four cycles of PRRT, with intervals of 6–8 weeks (PSMA) or 6–12 weeks (PRRT) between cycles 14 15 . Both approved radiopharmaceuticals, such as Pluvicto and Lutathera, and in-house preparations manufactured under GMP conditions are established in clinical practice 16 17 . Consequently, individual discharge decisions must incorporate not only the most recent administration but also the expected cumulative dose from all planned and already completed cycles within the same calendar year, with probably a mixture of different radiopharmaceuticals used.

This regulatory framework underscores the importance of estimating the effective half-life of ^177^ Lu in individual patients to enable reliable dose calculations. The effective half-life, determined by the combined influence of physical decay, tumor burden, and biological clearance, governs the temporal decline of emitted radiation and thereby the potential external exposure to third parties following patient discharge.

In the currently still predominantly palliative setting, in which ^177^ Lu-based therapies are administered, patient acceptance is strongly influenced by the perceived safety, time spent in the hospital and transparency of treatment. Establishing an evidence-based foundation for discharge decision-making is therefore essential. Systematic collection and evaluation of clinical and procedural data could provide the basis for a comprehensive safety concept, which in turn may serve as a formal reference for regulatory approval. Such a framework would not only strengthen the legitimacy of therapeutic practice but also improve transparency and foster trust among patients, caregivers, and stakeholders.

Therefore, the present retrospective multicenter study aims to analyze ^177^ Lu-based therapeutics in clinical routine across several German nuclear medicine departments, with a focus on estimating the effective half-life. Based on this analysis, it is anticipated that recommendations for effective half-life values can be given to support assessments relevant to radiation protection. Specifically, this retrospective study examines the measurement techniques, modelling approaches, and institutional thresholds employed to quantify radiation exposure to third parties and to guide discharge decisions.

## Materials and Methods

This retrospective multicenter study was conducted across eight nuclear medicine departments from seven different federal states in Germany, including university hospitals and municipal institutions. All participating centers routinely perform inpatient therapies with ^177^ Lu-labelled radiopharmaceuticals.

### Study Design and Ethical Approval

Institutional procedures related to patient discharge following ^177^ Lu therapy were compared across centers using a structured data collection template. The study was approved by the lead Ethics Committee of University Hospital Jena reference number: No. 2024–3421.

### Classification by Radiopharmaceutical Type

To account for pharmacokinetic variability, centers differentiated on the type of radiopharmaceutical used:

- Commercial products:
  1. Pluvicto = [ ^177^ Lu]Lu-PSMA-617 (Pluvicto, Novartis AG, Basel, Switzerland)
  2. Lutathera= [ ^177^ Lu]Lu-DOTATATE (Lutathera, Novartis AG, Basel, Switzerland)
- In-house preparations (using GMP-grade kits and in-house radiolabelling):
  1. ihPSMA = [ ^177^ Lu]Lu-PSMA-I&T
  2. ihRRT = [ ^177^ Lu]Lu-DOTATOC

Pharmacokinetic differences, especially in terms of plasma clearance and tumor retention, were not considered when analyzing dose rate decay and estimating T _1/2, eff_ .

### Dose Rate Measurement and Kinetic Modeling

Each center reported their local standard operating procedures for calculating the T _1/2, eff_ , with dose rate measurements (DRM) or gamma camera measurements and the subsequent discharge planning, including the following informations:

- Type of therapy
- Type and calibration status of measuring device for discharging (handheld or fixed/wall mounted)
- Geometric setup (distance from patient, anterior/posterior positioning)
- Further measurements, conducted using a gamma camera
- Timing and frequency of measurements

### Half-life Determination

Measurements of the dose rate were retrospectively evaluated in eight clinics. For this purpose, depending on the data available, different time points were considered. Subgroup separation was achieved through the implementation of a measurement time interval. Some clinics measured the patients until < 48 h after administration due to discharging. In order to apply a uniform adjustment model based on the raw data obtained, mono-exponential functions were fitted to three consecutive measurement points for the calculation of effective half-lives; an early T _1/2, eff_ with the last measurement point after two days (< 48 h) and a late T _1/2, eff_ with the last measurement point between 48 to 168 h (< 168 h). The median and 90 ^th^ percentile of the effective half-lives for each treatment type were calculated. The 90 ^th^ percentiles can be taken as practical estimators for the effective half-life when calculating patient discharge times and the resulting effective dose for other persons, see below.

The results were subdivided by treatment type, with the estimates of third-party exposure compared based on physical and T _1/2, eff_ .

### Statistical Analysis

The mean value, along with the standard deviation, median, range, and 90 ^th^ percentile, was calculated for each radiopharmaceutical and time interval. To ascertain whether the effective half-life differed significantly depending on the fitting model (<48 h vs. < 168 h), intraindividual comparisons were performed separately for each radiopharmaceutical and then examined for significant differences. Given the non-normal distribution of the data, the non-parametric Mann-Whitney-U-test was administered. The level of significance was defined as α = 0.05 . All analyses were conducted using the R programming language (Version 4.4.1, R Core Team, Vienna, Austria) and Integrated Development Environment RStudio (Version 2024.12.0, Posit-Software, PBC).

## Results

All participating centers contributed a total of n=210 sets of dose rate measurements, divided into n=25 ihRRT, n=116 ihPSMA, n=14 Lutathera, and n=55 Pluvicto datasets following the administration of ^177^ Lu-labelled radiopharmaceuticals including the dose rate measurements ( Table 1 ). The mean activity of ^177^ Lu regardless of therapy type was (7149 ± 522) MBq (median: 7399 MBq, range: 5077–7853 MBq). Two centers used handheld devices, three employed fixed mounted and another three ceiling-mounted detectors. Measurement distances ranged from 1 to 2 m, with most centers opting for a 2 m anterior setup. Three centers performed both anterior and posterior measurements for calculating a mean of both or used additional information from whole body scans using gamma cameras. The number of measurements per patient ranged from 3 to 7, timing varied considerably: while some centers employed fixed daily schedules (e.g., 9:00, 13:00 and 20:00), others defined time points relative to the actual administration (e.g., 12 h, 36 h, 48 h p.i.). Three centers routinely conducted measurements later than 48 h after administration, reflecting logistic limitations related to the discharge workflow. A small number of outliers were identified, and following individual case review, it was determined that these were attributable to tumor burden.

**Table:** **Table 1** Clinical site comparison for intra-therapeutic dose rate measurements.

Site Nr.	Type	DRM type	DRM status	Distance patient to DRM	Method of measurement	Time-points p.i.						
	1 = ihPSMA 2 = Pluvicto 3 = ihRRT 4 = Lutathera		oc = officially calibrated certified by authorities ivc = inhouse verication and calibration		a = anterior p = posterior s = standing l = lying	0–6 h	12–20 h	24–30 h	36–42 h	44–48 h	72–96 h	144–168 h
1	1, 3	mounted Berthold LB 123	oc	2 m	a + p/s	x	x	x	x	x	x	x
2	1, 3	mounted Berthold LB 123	ivc	2 m	a + p/s	x		x		x		
3	1	handheld Thermo Scientific FH-40+	ivc	2 m	a/s			x		x	x	x
4	1, 2, 3, 4	wall mounted Step Sensor DLMon	ivc	2 m*	a/l	x	x	x	x	x		
5	1, 2, 3	wall mounted Step Sensor DLMon	ivc	2 m*	a + p/l	x	x	x	x	x		
6	1	wall mounted Automess 6150AD 18+	ivc	2 m*	a/l	x	x	x	x	x		
7	2, 3, 4	Mounted Berthold UMO-LB123+	oc	2 m	a/s			x		x		x
8	2, 3, 4	handheld Thermo Scientific FH-40	oc	2 m	a + p/s	x		x		x		
* The actual distance between the ceiling-mounted measuring device and the patient is approximately 2.6 m. During commissioning, calibration and correction was performed for distances up to 2.0 m. An annual calibration control was performed. + Only DRM measurements performed for discharge, separate time-point measurements for calculating the effective half-life with gamma camera measurements.												

**Fig. 1:**
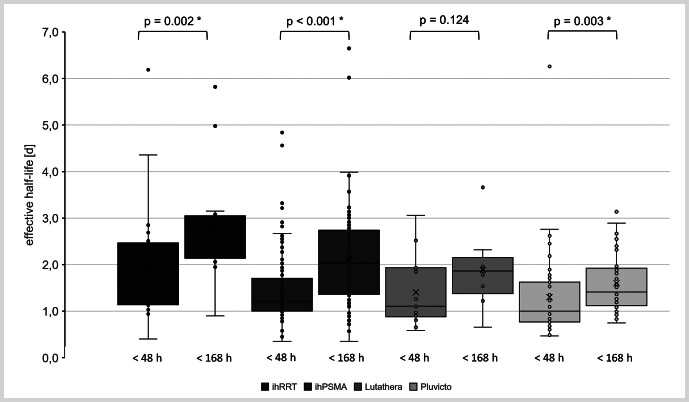
Effective half-lives in days of the four radiopharmaceuticals divided in measurements < 48 h and < 168 h.

For time windows < 48 h ( Fig. 1 ), the effective half-life showed the following distributions: Pluvicto exhibited the shortest mean effective half-life of (1.30 ± 0.91) d (median: 1.00 d; range: 0.47 – 6.26 d; 90 ^th^ percentile: 2.35 d), followed by Lutathera with (1.40 ± 0.75) d (median: 1.11 d; range: 0.58 – 3.06 d; 90 ^th^ percentile: 2.35 d), ihPSMA with (1.45 ± 0.76) d (median: 1.20 d; range: 0.35 – 4.84 d; 90 ^th^ percentile: 2.58 d), and ihRRT with (1.97 ± 1.23) d (median: 1.67 d; range: 0.40 – 6.19 d; 90 ^th^ percentile: 2.44 d).

In contrast, for time windows < 168 h ( Fig. 1 ), higher mean effective half-life were observed across all groups: Pluvicto reached (1.60 ± 0.63) d (median: 1.41 d; range: 0.75 – 3.14 d; 90 ^th^ percentile: 2.57 d), Lutathera showed (1.89 ± 1.14) d (median: 1.86 d; range: 0.66 – 3.66 d; 90 ^th^ percentile: 2.59 d), ihPSMA reached (2.10 ± 0.98) d (median: 2.04 d; range: 0.35 – 6.65 d; 90 ^th^ percentile: 3.08 d), and ihRRT demonstrated the longest values with (2.85 ± 1.12) d (median: 2.72d; range: 0.90 – 5.82 d; 90 ^th^ percentile: 3.88 d).

Significant differences in T _1/2, eff_ were observed between several therapy types and measurement time intervals ( Table 2 ). Notably, comparisons involving Pluvicto < 48 h and ihRRT < 168 h with other subgroups yielded the lowest p-values, indicating pronounced variability as a function of both therapy and timing.

**Table:** **Table 2** P-values from pairwise comparisons between therapy and measurement time intervals (< 48 h vs.  < 168 h) based on effective half-life.

	ihRRT < 48 h	ihRRT < 168 h	ihPSMA < 48 h	ihPSMA < 168 h	Lutathera < 48 h	Lutathera 168 h	Pluvicto < 48 h	Pluvicto < 168 h
ihRRT < 48 h	–	0.002*	0.018*	0.187	0.854	0.728	0.001*	0.294
ihRRT < 168h	–	–	< 0.001*	0.007*	< 0.001*	0.010*	< 0.001*	< 0.001*
ihPSMA < 48 h	–	–	–	< 0.001*	0.549	0.038*	0.035*	0.072
ihPSMA < 168h	–	–	–	–	0.003*	0.569	< 0.001*	0.003*
Lutathera < 48 h	–	–	–	–	–	0.124	0.418	0.190
Lutathera < 168h	–	–	–	–	–	–	0.019*	0.263
Pluvicto < 48 h	–	–	–	–	–	–	–	0.003*
Pluvicto < 168h	–	–	–	–	–	–	–	–
*Statistically significant differences (p < 0.05)								

**Fig. 2:**
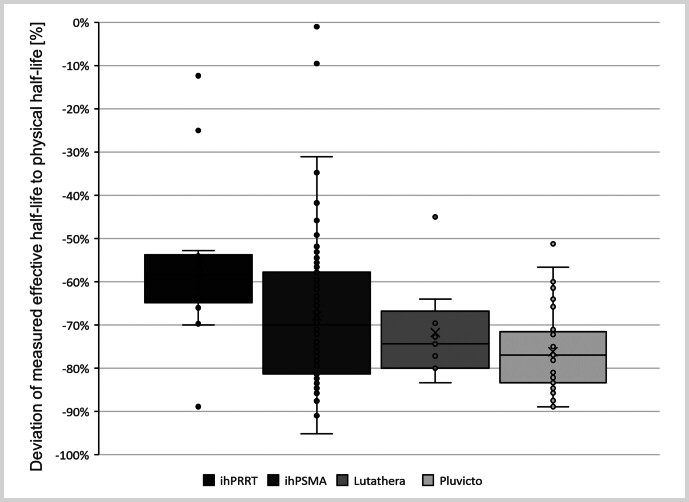
Deviation of the measured effective half-lives from measurements < 168 h compared to the physical half-lives (T _eff_ ).

Fig. 2 shows that the T _1/2, eff_ , which had been chosen with regard to the lowest number of datasets for one of the radiopharmaceuticals investigated in this work to provide a statistically sound basis for generic estimators, of all compounds was substantially lower than the T _1/2, phy_ of ^177^ Lu. The median deviation ranged from –54 % to –74 %, with the smallest median deviation observed for ihRRT (–54 %), followed by ihPSMA (–67 %), Lutathera (–72 %), and Pluvicto (–74 %).

Based on the 90 ^th^ percentiles of the measured T _1/2, eff_ , the following values are recommended as conservative estimates for radiation protection and dose calculation for third parties as also shown in Table 3 : 4.0 d for ihRRT, 3.5 d for ihPSMA, 3.0 d for Lutathera, and 2.0 d for Pluvicto. These values reflect upper-range kinetics in 90 % of treated patients and can be used to estimate expected dose exposures in non-monitored environments.

**Table:** **Table 3** Estimated cumulative exposure for different numbers of therapy cycles per year based on dose rate and time of discharge.

Type	Recommended as conservative estimates for Teff [d]	Follow-up dose for third parties per application [mSv]	Max. number of therapies per year for exposure < 1 mSv for third parties
ihRRT	4.0	0.31	3
ihPSMA	3.5	0.17	5
Lutathera	3.0	0.10	10
Pluvicto	2.0	0.07	13
all*	3.0	0.12	8
* mixture of different Types			

## Discussion

One of the main findings of this study is the substantial heterogeneity in dose rate measurement protocols among the participating centers. Differences were observed in terms of measurement devices, source-detector distances, anatomical orientation (anterior vs. posterior), frequency, and timing of measurements. While some of this variability reflects local infrastructure and logistical constraints, it also introduces inconsistencies in the estimation of patient-specific T _1/2, eff_ and, consequently, in the extrapolated exposure to relatives and caregivers. However, no statistically significant differences were observed between the facilities in terms of the half-life determined. This can be attributed to the fact that, even in cases where a system is not calibrated, the temporal component, i.e. the T _1/2, eff_ , can be accurately determined provided the same setup is utilized for all patient-specific measurements.

From a methodological point of view, it is important to differentiate between absolute measurement accuracy and relative measurement consistency. While the former is essential when comparing dose rates across institutions or against regulatory thresholds, the latter is sufficient for modeling the time-dependent decline in dose rate. This, for instance, applies for estimating the T _1/2, eff_ provided that all measurements in a given patient are performed under identical geometric and instrumental conditions. In such cases, the lack of calibration only affects the absolute values, not the temporal decay pattern, allowing for reliable curve fitting and relative kinetic interpretation.

Bi-exponential modeling of dose rate decay is a well-established approach for determining T _1/2, eff_ , especially when both fast and slow clearance components are relevant. Particularly in cases of high tumor retention with a long half-life, pronounced bi-exponential kinetics may occur, which cannot be adequately represented by a simple exponential function and should be fit accordingly. However, the accuracy and stability of the fitted parameters are strongly dependent on the availability of late-phase data points. This underlines the importance of integrating at least one data point in the terminal elimination phase to avoid over- or underestimation of residual activity and associated dose rates. Given the frequent discharge of patients after 48 h in Germany, this poses a methodological challenge and supports the development of standardized fitting protocols, potentially supplemented by population-based decay constants derived from larger datasets.

Although this variability did not universally reach statistical significance, it is clinically relevant in the context of radiation protection, as it affects the predictability of external dose contributions and thus the precision of discharge planning.

A key finding of this multicenter study is the consistent observation that the T _1/2, eff_ derived from patient-specific dose rate measurements are, on average, substantially shorter than the T _1/2, phy_ of 6.7 d. This effect was observed across all therapeutic administrations and reflects the influence of their pharmacokinetic properties.

To quantify the effect of observation duration on calculated half-life, intraindividual comparisons were performed between measurements taken within the site performing measurements during the first 48 h and those extending measurement points beyond 48 h after administration. Mann-Whitney-U-tests revealed statistically significant differences in T _1/2, eff_ between these time intervals for ihRRT (p = 0.002), ihPSMA (p < 0.001) and Pluvicto (p = 0.003). Only for Lutathera the difference did not reach statistical significance (p = 0.124). These results support the notion that shorter acquisition windows (< 48 h) systematically underestimate the T _1/2, eff_ , particularly for radiopharmaceuticals with slower tissue clearance.

Given this, the use of early time-point measurements alone may be insufficient for accurate dosimetric estimation or radiation protection assessment. Nonetheless, the consistent deviation pattern across compounds may permit conservative extrapolation using percentile-based half-life estimates, as proposed in this study.

This observation is physiologically plausible, as the T _1/2, eff_ incorporates both physical decay and biological clearance mechanisms – primarily renal excretion and, to a lesser extent, hepatobiliary elimination. Consequently, assuming a purely physical decay model would systematically overestimate the exposure of third parties and lead to overly conservative discharge planning. The recommended half-lives show that, except for ihRRT and very close to the ihPSMA, the other preparations allow the maximum number of therapy cycles according to the guidelines 1 mSv per year for the public depending on the release time. This would not be the case when only using basic T _1/2, phy_ . Considering the variability in delivery conditions, necessary activity adjustments, and patient-specific circumstances, it may in practice become necessary to combine different radiopharmaceuticals in one period of cycles (e.g., ihPSMA with Pluvicto, or ihRRT with Lutathera). This clinical reality further underscores the need for defining a combined recommended half-life value, which would provide a more robust and universally applicable basis for both clinical decision-making and regulatory frameworks. Due to the multicenter retrospective data situation, data from patients with higher tumor volumes could not be explicitly evaluated and analyzed. This was not explicitly taken into account during data collection. One limitation at that point was that the maximum cycles per year were conservatively estimated based on constant uptake and T _1/2, eff_ in all cycles, which is unlikely to be the case under real-world conditions. The finding supports the clinical relevance of individualized or protocol-based T _1/2, eff_ estimation to improve the precision of dose calculations, without compromising safety. In routine settings, using institution-specific or population-based T _1/2, eff_ values derived from validated measurements could provide a pragmatic and evidence-based alternative to worst-case assumptions based solely on physical decay.

The proposed extrapolated T _1/2, eff_ are not intended to replace individualized intratherapeutic measurements, which remain essential for accurate patient-specific dosimetry and radiation protection. To better assess the cumulative exposure potential for third, extended dose rate measurements beyond the current standard – ideally up to 14 d post- administration – may offer valuable insights into the long-term effective clearance behavior of ^177^ Lu labelled radiopharmaceuticals. First approaches already exist for PRRT 18 . Such data would allow for a more accurate risk assessment of residual external and internal radiation burdens within the home environment, where patient movement, proximity, and shielding conditions vary greatly. When combined with the results of the present study, these extended measurements could serve as the foundation for a differentiated risk-based approach to discharge planning.

In addition to the patient’s external radiation exposure, the excretion of the administered activity must also be taken into account as part of the hospitalization decision. High renal elimination is characteristic of almost all low-molecular compounds and peptide therapeutics, which leads to a correspondingly high concentration of activity in the urine. The activity that is not bound in organs and tumours leaves the body as a fast clearance component with a half-life of a few hours, meaning that 70% or more is often excreted after one day 12 19 . However, depending on the percentage of bound activity and the half-life of the slow clearance component, more than half of the activity may still be excreted after 48 hours in individual cases in patients with high tumour uptake. Especially in patient populations with metastatic castration-resistant prostate cancer (mCRPC), contamination events through urine have been documented in the clinical setting, as documented by recent conference reports, among others 13 19 . Such contamination poses the risk of significant local skin doses, which can occur in particular in direct contact with contaminated surfaces or materials and are described in the literature with individual doses in the range of several 10 mSv 19 20 . In addition to the limitation of the effective dose of 1 mSv for third parties required in § 80 StrlSchG, compliance with the limit value of 50 mSv for the skin dose is therefore also coming into focus. Renal excretion is less relevant for antibody-based radiopharmaceuticals, so that this risk is comparatively reduced. Nevertheless, this aspect underlines the need to consider not only the external radiation exposure, but also the contamination and skin dose risk in a differentiated manner when assessing the discharge criteria.

Therefore, rather than aiming for rigid national thresholds or singular discharge values, a more productive approach may lie in harmonizing the methodology:

- Defining minimum measurement requirements (e.g., ≥ 3 time points, ideally with one > 48 h post-administration)
- Standardizing geometric conditions
- Providing recommended half-life for different therapies, stratified by radiopharmaceutical

Such an approach would preserve flexibility for clinical workflow, while ensuring regulatory compliance and enhancing radiation protection by basing assessments on real patient kinetics rather than theoretical decay models.

## Conclusion

This multicenter study demonstrates that the T _1/2, eff_ of ^177^ Lu-based therapies is significantly shorter than the T _1/2, phy_ of ^177^ Lu, with median deviations ranging from –54 % to –74 % depending on the radiopharmaceutical. An important implication of our findings is that post 48 h measurements, which showed comparatively higher values of half-life, should be integrated into future dose estimations to improve accuracy and clinical relevance. In the future, patient-specific measurement of half-life remains “gold standard”; alternatively, the use of population-based half-life can be an alternative for discharge planning with lesser effort but in many cases longer assumed half-life compared to measured half-life. However, in clinical scenarios where direct measurement is not feasible, reference values based on the 90 ^th^ percentile of the T _1/2, eff_ distribution – stratified by therapeutic indication – may provide a conservative yet pragmatic framework to ensure compliance with dose limits, such as the 1 mSv/year threshold for third-party exposure. Nevertheless, it must be emphasized that these extrapolated values cannot substitute for individualized intratherapeutic assessment. Further studies for long-term kinetic measurements beyond 1–2 weeks, including different patient subgroup parameter (e.g. the glomerular filtration rate, tumor load) are warranted.

## Abbreviations

a: anterior
DRM: Dose Rate Meter
GMP: Good Manufacturing Practice
ihPSMA: In-house Lu-177-PSMA-I&T
ihRRT: In-house Lu-177-DOTATOC
ivc: inhouse verication and calibration
l: lying
Lu-177: Lutetium-177
Lutathera: Lu-177-DOTATATE (Fa. Novartis)
mSv: Millisievert
n: sample size
oc: officially calibrated certified by authorities
p: posterior
p.i.: post injectionem
Pluvicto: Lu-177-PSMA-617 (Fa. Novartis)
PSMA: Prostate-Specific Membrane Antigen Therapy
RRT: Receptor Radionuclide Therapy
s: standing
StrlSchV: Strahlenschutzverordnung (German Radiation Protection Ordinance)
µSv/h: Mikrosievert per hour

## References

[1] Sartor O de Bono J Chi KN Lutetium-177-PSMA-617 for Metastatic Castration-Resistant Prostate Cancer N Engl J Med 2021 385 1091 1103 10.1056/NEJMoa2107322 34161051 PMC8446332

[2] Strosberg J El-Haddad G Wolin E Phase 3 Trial of (177)Lu-Dotatate for Midgut Neuroendocrine Tumors N Engl J Med 2017 376 125 135 10.1056/NEJMoa1607427 28076709 PMC5895095

[3] Wanke C Borowski M Freudenberg R Vorgehensweisen zur Einhaltung der Entlassungskriterien bei Patienten nach nuklearmedizinischer Therapie. PROCEDURES FOR COMPLIANCE WITH RELEASE CRITERIA FOR PATIENTS UNDERGOING NUCLEAR MEDICINE THERAPIES Berlin 2024

[4] Kranert W Sabet A Bockisch B Berechnung des Entlassungsgrenzwertes für Patienten nach Therapie mit Lu-177-PSMA und -DOTATATE Nuklearmedizin 2019 58 10.1055/s-0039-1683697

[5] Bundesministerium für Umwelt NuR. Richtlinie zur Strahlenschutzverordnung (StrlSchV) – Fachliche Weisungen 2011

[6] Kühnel C Freesmeyer M Seifert P Radioiodine therapy of benign thyroiddisease Angewandte Nuklearmedizin 2023 46 38 50 10.1055/a-1802-8333

[7] Strahlenschutzkommission (SSK). Bewertung von Radionukliden zur Anwendung in der systemischen nuklearmedizinischen Therapie. Verabschiedet in der 321 Sitzung der Strahlenschutzkommission am 22/23 September 2022 2022

[8] Rodriguez JC BioChroma – A New and Patented Technology for Processing Radioactive Wastewater from Nuclear Medicine Therapy Facilities in Hospitals and Clinics World J Nucl Med 2012 11 12 18 10.4103/1450-1147.98735 22942776 PMC3425222

[9] Zippel C Giesel FL Kratochwil C PSMA radioligand therapy could pose infrastructural challenges for nuclear medicine: results of a basic calculation for the capacity planning of nuclear medicine beds in the German hospital sector Nuklearmedizin 2021 60 216 223 10.1055/a-1351-0030 33530110

[10] Kuhnel C Winkens T Niksch T Design and practical evaluation of a shielded application system for intravenously administered radionuclide therapies Nuklearmedizin 2020 59 323 331 10.1055/a-1131-7558 32193882

[11] Fitschen J Knoop BO Behrendt R External radiation exposure and effective half-life in Lu-177-Dota-Tate therapy Z Med Phys 2011 21 266 273 10.1016/j.zemedi.2011.05.001 21719263

[12] Kurth J Krause BJ Schwarzenbock SM External radiation exposure, excretion, and effective half-life in (177)Lu-PSMA-targeted therapies EJNMMI Res 2018 8 32 10.1186/s13550-018-0386-4 29651569 PMC5897276

[13] Kranert WT Bockisch B Wichert J Anticipating Criteria for Discharge after Lu-177-PSMA Treatment – Discussion of Several Scenarios Nuklearmedizin 2022 61 111 119 10.1055/a-1697-8126 35170005

[14] Ladriere T Faudemer J Levigoureux E Safety and Therapeutic Optimization of Lutetium-177 Based Radiopharmaceuticals Pharmaceutics 2023 15 10.3390/pharmaceutics15041240 PMC10145759 37111725

[15] Kratochwil C Fendler WP Eiber M Joint EANM/SNMMI procedure guideline for the use of (177)Lu-labeled PSMA-targeted radioligand-therapy ((177)Lu-PSMA-RLT) Eur J Nucl Med Mol Imaging 2023 50 2830 2845 10.1007/s00259-023-06255-8 37246997 PMC10317889

[16] Hennrich U Eder M [(177)Lu]Lu-PSMA-617 (Pluvicto(TM)): The First FDA-Approved Radiotherapeutical for Treatment of Prostate Cancer Pharmaceuticals (Basel) 2022 15 10.3390/ph15101292 PMC9608311 36297404

[17] [Anonym] Lutetium lu 177 dotatate (Lutathera) for gastroenteropancreatic neuroendocrine tumors Med Lett Drugs Ther 2018 60 e152 e153 30383735

[18] Gleisner KS Brolin G Sundlov A Long-Term Retention of 177Lu/177mLu-DOTATATE in Patients Investigated by gamma-Spectrometry and gamma-Camera Imaging J Nucl Med 2015 56 976 984 10.2967/jnumed.115.155390 25999429

[19] de Bakker M Dominicus N Meeuwis A Urinary excretion kinetics of [(177)Lu]Lu-PSMA-617 Eur J Nucl Med Mol Imaging 2023 50 3572 3575 10.1007/s00259-023-06328-8 37421427 PMC10547615

[20] Brosch-Lenz J Nekolla S Tischendorf L Evaluation of the contamination risk during 177Lu-PSMA therapy of advanced prostate cancer Journal of Nuclear Medicine 2023 64 P870

